# Little Helps

**Published:** 1906-12-15

**Authors:** George Zederbaum

**Affiliations:** Charlotte, Mich.


### LITTLE HELPS.

GEORGE ZEDERBAUM, D. D. S., CHARLOTTE, MICH.

Below are a dozen hints that I can recommend. With me they are all original. Perhaps I stole some-one's idea; no doubt I have, as there is nothing new under the sun; but lots of times ideas get pretty old before we get hold of them.

There is one thing that I aim to practice: whenever in the course of my work I happen across something that looks valuable and reasonable I jot it down immediately on my memorandum pad, then I try it several times as the opportunity affords. In this manner I have tried out the few hints I give and I am satisfied they will please.

When polishing posterior fillings, to avoid scratching mirror and to keep tongue and cheeks away while so doing I find nothing better than a medium sized tea-spoon. Insert it always with the concave side toward the surface to be polished and you will soon experience the ease with which you can accomplish your work and will be surprised at almost complete shutting out of saliva.

A magnet is a very useful article at the chair. How often a broach drops on the floor, or some part of your midget separator, or several burs? Magnet will find these when you can't. Time is saved and the temperature is kept at the normal.

If perchance you overheated your flask and some melted wax has escaped into the water, to rid the water of floating wax, remove the flask and dip into the water a bunch of tissue paper, and with sort of swabbling motion go over its surface, the water will be cleared.

Always weigh your gas cylinders before and after administration . You will then know first, how much you have given your patient: second, whether there was any leakage; third, whether you have enough left to commence and finish another administration without perhaps being obliged to run to another dentist to borrow his cylinder to complete your operation, even his may then be found nearly empty.

When grinding ready made porcelain crowns to fit the root, be sure and repolish them as best you can. Patients soon learn the difference.

When the ligature fails to do its duty, slips incisally especially so in the lower anterior teeth, double it and then pass around, and many times it will stay where put.

After temporary stopping has been inserted in proximal cavity take a fine polishing strip, moisten with eucalpytol and pass into the embrasure to and fro several times. Filling will become smooth and its better retention is insured.

In extractions under general anaesthetics, I like corks better than the unsightly rubber blocks or even the metallic props. A lot of different sized corks at almost no cost, to be used but once and then burned is preferable in my estimation—they are liable to break and the pieces be inhaled by patient—Ed. Tie a piece of stout twine around the cork you selected to use, place it and your patient's teeth will rest comfortably and they will not bite through involuntarily.

Don't subscribe for too many dental journals; but be sure and read all there is in every one you do get. Don't lay it aside saying "I'll read that next week" for you might have to make over a plate just then, and first you know a new issue comes and you have not read the last month's number, and perhaps never will.

When leaving office for the night, be sure and turn off the floor or wall valve supplying your cuspidor. These sultry days sweat and rust the nickeled parts and wet and stain your floor.

Brush amalgam off your pluggers immediately after using. After it sets you can not well remove it without dulling the serrations.

A few drops of Compound Tincture of Benzoin, rubbed on your extracting hand will prevent the forcep-handles from slipping which they do many times on sultry and muggy days we all enjoy so.

